# StemBANCC: Governing Access to Material and Data in a Large Stem Cell Research Consortium

**DOI:** 10.1007/s12015-015-9599-3

**Published:** 2015-05-30

**Authors:** Michael Morrison, Christine Klein, Nicole Clemann, David A. Collier, John Hardy, Barbara Heiβerer, M. Zameel Cader, Martin Graf, Jane Kaye

**Affiliations:** HeLEX - Centre for Health, Law and Emerging Technologies, Department of Public Health, University of Oxford, Old Road Campus, Oxford, OX3 7LF UK; Institute of Neurogenetics, Department of Neurology, University of Lüebeck, Lüebeck, Germany; Hoffman-La Roche Ltd, Pharma Research and Early Development (pRED), Basel, Switzerland; Eli Lilly and Company, Erl Wood Manor, Windlesham, Surrey, UK; Institute of Neurology, Department Molecular Neuroscience, University College London, London, WC1N 1PJ UK; Concentris Research Management GmbH, Ludwigstr 4, D-82256 Fürstenfeldbruck, Germany; Nuffield Department of Clinical Neurosciences, University of Oxford, John Radcliffe Hospital, Oxford, OX3 9DU UK; The Weatherall Institute of Molecular Medicine, University of Oxford, John Radcliffe Hospital, Oxford, OX3 9DS UK; Department of Physiology, Anatomy and Genetics, MRC Functional Genomics Unit, University of Oxford, South Parks Road, Oxford, OX1 3PT UK

**Keywords:** Stem cell research (SCR), Governance, Materials and data access, Induced pluripotent stem cells (iPSCs), Research consortia, Innovative medicines initiative (IMI)

## Abstract

This paper makes the case for implementing an internal governance framework for sharing materials and data in stem cell research consortia. A governance framework can facilitate a transparent and accountable system while building trust among partner institutions. However, avoiding excessive bureaucracy is essential. The development and implementation of a governance framework for materials and data access in the *Stem cells for Biological Assays of Novel drugs and prediCtive toxiCology* (StemBANCC) consortium is presented as a practical example. The StemBANCC project is a multi-partner European research consortium, which aims to build a resource of 1,500 well characterised induced pluripotent stem cell (iPSC) lines for in vitro disease modelling and toxicology studies. The project governance framework was developed in two stages. A small working group identified key components of a framework and translated the project legal agreements into a draft policy document. The second phase allowed input from all consortium partners to shape the iterative development of a final policy document that could be agreed by all parties. Careful time management strategies were needed to manage the duration of this component. This part of the process also served as an exploratory space where different options could be proposed, potential gaps in planning identified, and project co-ordination activities specified.

## Introduction

Over the past decade there has been a significant rise in the use of public-private funding agreements to bring together academic and industrial organisations in large consortia. [1] One factor driving the creation of these research consortia is the increasing volume and complexity of datasets involved in addressing major challenges in the life-sciences. [2] Such volumes of data are often beyond the capacity of any one group or institution to process efficiently. Multi-sector collaborations are needed to leverage the infrastructure, multi-disciplinary expertise and other resources required to address these challenges. [3] Rising healthcare costs and declining returns from traditional pharmaceutical industry R&D are also driving support for academic-industry consortia dedicated to reinvigorating drug discovery through the application of novel technologies such as genomics and stem cell science. [3–5] Examples include the Québec Consortium for Drug Discovery (CQDM) in Canada and the Innovative Medicine’s Initiative [6] (IMI) in Europe. The need for collaborative sharing of biological samples and data on a large scale is also a key part of Europe’s agenda on addressing rare disease (RD) [7].

Sharing data, especially sensitive ‘individual level’ medical, personal, and biological data, requires appropriate governance. [8, 9] Governance incorporates the full range of formal and informal options for organising, co-ordinating and controlling activities and is not limited to the legal requirements of formal regulation. Regulatory requirements remain mandatory and must be met, but the legal requirements for SCR are adequately addressed elsewhere [10] and the focus here is on the less formal mechanisms of governance that can be applied to the internal organisation of a collaborative consortium. Having an internal governance system for data sharing between consortium partners can facilitate transparency and accountability, foster trust between institutions, and augment project organisation and co-ordination capabilities. However, governance systems also need to avoid unintentionally creating new obstacles to data sharing such as excessive bureaucracy and overly elaborate access procedures. A number of recent publications have discussed the challenges and benefits of implementing governance structures for data sharing within contemporary large consortia, but these focus predominantly on sharing of genomic data [8, 11, 12].

Although access to materials and data has been identified as especially critical to stem cell science [13] it has been suggested that the stem cell community has been somewhat slower to respond to the changing frameworks of biomedical innovation and translation. [4] Unlike genomic and other medical data, which can readily be digitised and shared through Information Communication Technology (ICT) platforms, stem cell research (SCR) involves material biological objects, which can only be shared through physical transportation between locations and partners. SCR therefore at least potentially raises different and unique challenges for sharing material and data amongst large academic industry research consortia. One obvious issue is that novel platforms for collaborative data sharing such as Datashield [2] have not been designed with SCR in mind and may be less relevant for SCR consortia. This reflects a more general sense that data and materials sharing in SCR consortia has not, to date, received adequate attention, especially compared to similar examinations of genomics-based consortia.

In order to redress this situation, this paper describes the development and implementation of an intra-consortium governance mechanism for sharing materials and data in a contemporary IMI research consortium, *Stem cells for Biological Assays of Novel drugs and prediCtive toxiCology* (StemBANCC). This case selection provides an example of how abstract challenges of materials and data sharing can be addressed in practice, in a working SCR consortium. While the specifics of many of the challenges encountered in the StemBANCC consortium may be particular to this project, the nature of these challenges and the methods for resolving them can stand as a useful resource for current and future SCR consortia.

To provide appropriate context for this discussion it is first necessary to briefly review the purpose and components of a governance system itself and to provide some additional details on the nature of the StemBANCC consortium. The greater part of this paper will then describe the design and implementation of the StemBANCC Biorepository Materials and Data Access policy and the insights that can be drawn from this experience for future SCR consortia.

## The StemBANCC Project

As with all IMI projects StemBANCC is organised and funded through a public-private partnership between the European Union (EU) and the European Federation of Pharmaceutical Industries and Associations (Efpia). A key goal of StemBANCC is to generate a resource of 1,500 well-characterised induced pluripotent stem cells (iPSC) from 500 participants across a range of disease groups and healthy controls. [14] The consortium is not merely a large-scale collection project, but is also committed to utilising these iPSC lines to develop robust cellular models of disease and assay platforms suitable for screening potential new drug candidates. In order to manage this ambitious task, the StemBANCC consortium incorporates a range of disciplinary expertise across partners from the pharmaceutical industry, small and medium enterprises (SMEs), and academic research institutions across Europe. With 35 partner organisation spread across 10 different countries and a budget of 55.6 million Euros over 5 years, StemBANCC is one of the largest of the 40-plus IMI consortia established to date.

Intellectual property rights, competition and data protection laws can all limit access to cell lines. [13] Consortia supported through the IMI and similar initiatives are specifically designed to overcome these barriers. [15] Partners in a consortium like StemBANCC become legally bound to a shared set of rules on intellectual property rights, attribution of contributions to peer-reviewed publications, financial arrangements between partners, and other terms. The benefits of these agreements are evident when it comes to establishing data governance structures, as the terms under which data can be shared and the entitlements of all partner groups to the shared project material are agreed in advance. The StemBANCC project agreement specifically states that a ‘Biorepository Materials and Data Access Committee’ (BMDAC) with responsibility for ‘transparent and accountable research governance with regards to use of the samples [the iPSC lines]’ will be established and sets a date for the committee to be operational as a project deliverable.

The agreement also stipulates that the online Stem Cell Database (StemDb) platform [16] will be used for storing and sharing clinical, genomic and other project data in digitised form. All of this data is guaranteed to be accessible by all consortium partners under the terms of the project agreement, so in practice governing access to StemDB has largely involved creating different access rights for those responsible for uploading and editing data and those who only need to view it. StemBANCC iPSC lines are also stored in a central repository, the Human Biomaterials Resource [17] (HBRC) in Birmingham, UK. Much of the work reported here focused on creating a governance system to ensure fair and timely access to this shared material resource for all consortium partners. Implementing a governance structure still requires work because, while IMI terms set out clear rules on who is entitled to access shared data and materials in collaborative consortia, these rules still require interpretation as to *how* the specified goals will be achieved in practice.

## What is Governance and why is it Needed?

At a general level, governance can be described as ‘the intentional activity of attempting to control, order or influence the behaviour of others’. [18] Governance is different from regulation in that the latter is limited to maters of formal law and regulatory bodies with legally defined powers and scope. Instead governance encompasses a wider range of formal and informal methods of co-ordinating and organising activities. There are a number of advantages to establishing systemic governance of research. Co-ordinating and organising complex, distributed activities like stem cell research helps create standardisation and foster efficiency. In terms of agreeing and implementing standards there are some parallels between the role the International Stem Cell Initiative plays in co-ordinating SCR in laboratories at a global level and the function of a governance framework in an international consortium, albeit with the latter at a smaller scale and for a limited duration. A governance framework must clearly set out what must be done, how it should be done, the order in which procedures should be carried out, and where responsibility lies for particular tasks. [8] This facilitates transparency – everyone involved knows what the rules are and that they apply equally to all partners and accountability, helping to build trust between partners who may not have previously worked together. Having a governance framework also defines decision making roles and provides opportunities for different partners to have input in a co-ordinated manner1 allowing even large projects to be responsive to unanticipated issues and to manage uncertainty [19].

When developing a governance framework for internal materials and data sharing within the StemBANCC consortium, the field of biobanking provides a useful starting point. Extant biobank governance frameworks include formal regulatory documents and procedures, such as mandatory licenses and review from Research Ethics Committees, as well as less formal norms of working with tissue culture, guidelines on best practice, policy documents, input from leaders in the field and mechanisms such as project Data Access Committees [8]. The basic components of the StemBANCC materials and data sharing framework, such as a Data Access Committee and a materials and data access policy document, were adopted from this list. In addition, the development of a specific governance framework needs to be targeted to the specific task of sharing materials and data and must be proportionate in making sure the burden of governance in terms of time and workload does not outweigh the advantages of having a governance framework. [8] The way in which these principles were enacted in practice is illustrated in the next section describing the design and implementation of the StemBANCC materials and data access framework.

## Creating a Materials and Data Access Governance Structure for StemBANCC

For many participants in a consortium, developing a materials and data access policy will not be their primary responsibility and will not have high priority in their list of allocated tasks. With StemBANCC, a small, dedicated working group was set up to begin the process of creating a policy document by translating the terms of the project agreement into an outline policy. This draft document was then sent out to the representatives of each consortium partner and leaders of relevant working groups for feedback and review. This initiated a second stage of iterative development, where the policy document was revised by the working group and send out again for further rounds of feedback until a consensus policy document acceptable to all consortium partners was arrived at. The duration of the processes was managed by issuing strict deadlines for partners to respond to each new draft of the materials and data access policy and by using multiple communication tools, such as emails for routine written comments on a draft and teleconferences to bring multiple partners together when an issue required intra-consortium discussion.

### Step 1: Translating the Project Agreement to Draft Policy

In order to comply with the terms of the StemBANCC project agreement, practical governance arrangements needed to ensure that i) all partners had equal access to project stem cell lines and ii) these cell lines could be requested for both the agreed tasks set out in the agreed description of work (project use) and for partners’ own ‘in-house’ research purposes (research use).

This first objective set a clear limit on the scope for action of the Biorepository Materials and Data Access Committee. There were no grounds for refusing any partner access to any StemBANCC iPS cell line so the committee’s role was to facilitate access in a fair and transparent way, not to decide who had access and who did not. The BMDAC was therefore positioned as an intermediary between the physical storage site for StemBANCC iPSC and the various partner groups and laboratories across Europe entitled to request these lines. The role of the committee is to:

- Create a single contact point and a well-defined process for requesting cell lines, removing this administrative burden from staff at the central repository.
- Design and make available a simple mechanism (a request form) for all consortium partners to request StemBANCC cell lines.
- Review cell line request forms sent to the committee. Requests cannot be refused, but if a form is not filled in correctly or is unclear more information can be requested.
- Retain approved forms to generate an audit trail of which lines have been requested, by which partners and for which purposes, providing transparency and accountability.
- Foster transparency and trust by ensuring that industry and academic partners are equally represented in the BMDAC membership (along with representatives of the initial working group and the consortium management).

The latter point was achieved in practice by requiring members of the committee to be appointed by nomination, where each nomination had to be approved by a vote of the consortium’s General Assembly.

**Fig. 1:**
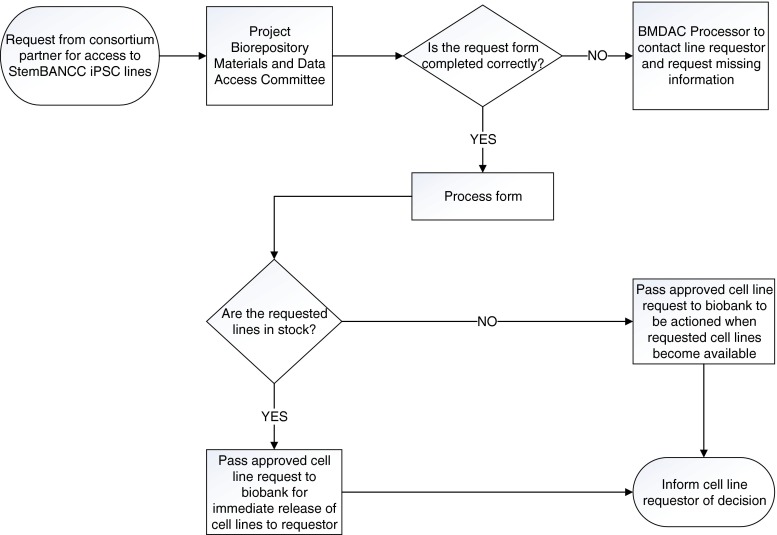
Basic governance framework for cell line access in StemBANCC

The second objective, allowing access for both project and in-house research raised the question of whether there was any benefit in establishing a practical difference between project and research usage. Both uses are legally guaranteed to partners under the project agreement, so it was important that neither use was subject to any undue restriction. However, a distinction would allow project work to be prioritized over research use if at any point a temporary shortage of any given iPSC line arose at the HBRC. Thus another component of application process emerged; two levels of access requests for consortium users – level 1 for project use and level 2 for research use. This basic governance process for accessing StemBANCC stem cell lines is illustrated in Fig. 1.

### Step 2: Iterative Development

Once the first draft policy document was written, it was then shared among consortium partners for review and feedback. This is a practical way of producing inclusivity by allowing all partners to have their say in producing an important decision making tool for the consortium. Unsurprisingly a commonly expressed concern was that increased bureaucracy risked impeding the scientific work. To address this, it was decided that there should be an agreed maximum time period between the BMDAC receiving a cell line request and either authorising the release of cell lines from the central repository or requesting further information. It was further proposed that this ‘turnaround time’ for cell line requests should be linked to the level of the request. Level 1 requests would be processed and a decision communicated to the requestor within a maximum of three working days while Level 2 requests, being slightly lower priority, would have a maximum turnaround time of five working days.

This iterative process for developing a policy allows some elements of the policy to become agreed at each iteration, permitting a general outline or skeleton of the proposed governance structure to emerge early on, while the finer details of each major component can be added with subsequent and more targeted iterations. For example, once a standard cell line request form was agreed as an appropriate tool, and a distinction between level 1 and 2 access requests accepted, the issue of specifically what information needed to be recorded about level 1 and 2 requests on the request form could be discussed. At one point a 300 word description of the intended research for which each cell line was being requested was mooted, but rejected on the grounds of being overly time consuming for requestors. A number of partners also felt that the requirement of an explicit description of ‘in house’ research projects associated with level 2 requests could compromise the privacy of the non-collaborative parts of their research. Consequently it was agreed that level 1 requests should list the project task (as set out in the original project documents) on the cell line request form, whilst level 2 requests would be characterised by selecting one or more elements from a pre-defined tick-box list, such as ‘pre-clinical research’ or ‘neuroscience’.

The second part of the development process also brings the majority of consortium partners into contact with the working group and with each other at a relatively early stage in the project. It is an opportunity for new or unexpected issues or gaps in existing planning to be uncovered and discussed. Issues that were raised in the course of the development of the StemBANCC BMDAC policy included, sharing lines with external partners later in the project, whether valuable tissue samples and data from external projects could be incorporated into the project, and whether, given the research use provisions, partners could include collaborations with third parties as part of their entitlement to use StemBANCC iPSC for in-house research.

Not every issue needs to be addressed in the context of discussing the data access policy and some can readily be moved to other fora within the project. Some are relatively simple to resolve; for example the issue of third party collaborations on non-project (level 2) research was addressed by adding a monitoring section on the cell line request form to check if level 2 uses involve any planned collaborations and imposing a requirement on the requesting party to state how StemBANCC material and IP will be protected in such instances.

Others are more complex and generate new project tasks and roles for governance actors. The idea of including ‘external’ lines via the standard means of a material transfer agreement (MTA), was deemed to be in direct conflict with the project’s intention of creating an open resource for European researchers, as an MTA could allow third parties ‘reach through rights’ that would limit the free use of on StemBANCC material. However some project partners were willing to share existing tissue samples with StemBANCC to be reprogrammed without incorporating any legal claims to the reprogramed material. This then led to an extra project task being established for the project’s ethics members in assessing the informed consent provisions of the external projects through which these tissue samples were collected and evaluating whether the provisions were adequate to permit the samples to be incorporated in StemBANCC. It also led to discussion of how these external lines fit into the StemBANCC governance framework and ultimately resulted in an additional category of ‘rare lines’ being developed.

**Fig. 2:**
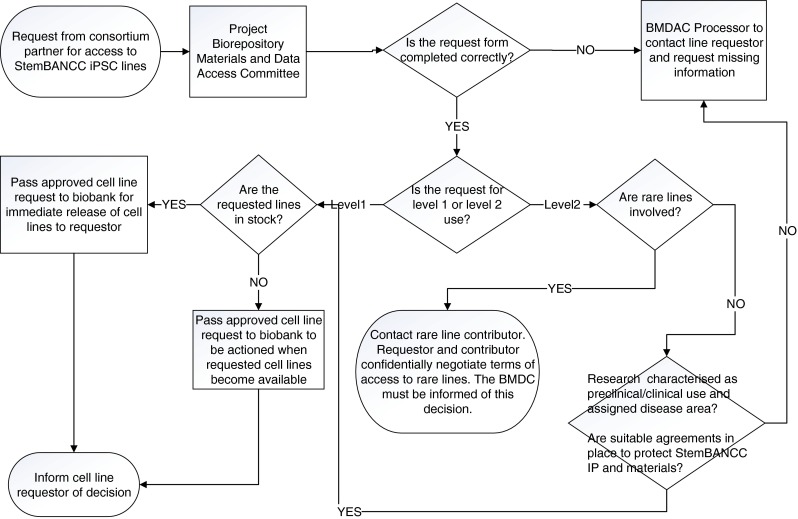
Operational governance framework for cell line access in StemBANCC

Rare lines described a small number of tissue samples with very rare genomic variants which could be shared with StemBANCC for project use, but where the ethics approval on the lines meant that the original custodian had to give explicit and specific permission for each use. In this situation the standard information provided for level 2 research use requests was insufficient to allow approval. The procedure developed to deal with this case was that level 2 requests for rare lines could be made, but the requestor and line custodian would then agree to discuss the details of proposed research in confidence before the custodian would make a final decision. Ultimately, the StemBANCC Biorepository Materials and Data Access policy that resulted from this iterative process was unanimously approved by a vote of the project General Assembly, which included voting representatives of all consortium partners. The operational version of the governance process for accessing StemBANCC stem cell lines is illustrated in Fig. 2.

## Conclusion

The processes and discussions described above cannot claim to be a complete account of everything that occurred during the creation of a materials and data access governance framework for StemBANCC. They are necessarily a selection designed to illustrate the type of issues involved in building a consortium-specific policy. As noted above these exact challenges are unlikely to recur in exactly the same way in other SRC consortia. Nonetheless, they do demonstrate the application of the key principles of better regulation - consistency, transparency, accountability, proportionality and targeting [8] in the practical development of a consortium materials and data access policy. There are also certain basic commonalities for all large SCR collaborative consortia. As material objects stem cells need to be stored at a physical location. Whether this is a single site for the whole project (as with StemBANCC) or multiple sites will depend on the needs of the project. However, there will still need to be a governance system in place to set out the procedures for accessing lines and to record the transfer of lines between sites for audit purposes. Aside from enabling transparency and accountability, there are clear advantages for reproducibility and reliability of research findings if different groups within a project working on the same line or lines are all working on samples from the same storage site, supplied at the same passage number. The legal agreements underpinning future academic-industry collaborations in SCR supported by the IMI and other initiatives may change but they will still require translation into practical arrangements of policy documents and procedures.

In this, the overall techniques applied in the creation of the StemBANCC BMDA policy are relevant to other current or future consortia. The use of a small working group to translate the requirements of a consortium agreement into a draft document for a governance structure is a practical solution to the problem that a general request for early input on data sharing policy is likely to result in vague or very general recommendations or a laissez-faire approach. At the same time, collaborations are inherently relational – they can only succeed if the different groups involved can agree to work in concert. A shared goal is an important unifying factor, but it needs to be complemented by internal mechanisms that generate interaction between partners. In governance terms this means taking a network approach to developing group documents and procedures rather than a top-down hierarchical approach, hence the second, iterative phase of policy development that allows all partners to be equally involved. This helps to build trust, which is especially important when partners are geographically separated, come from different institutional cultures and have different sets of expertise. It also has the merit that partners are more likely to feel a joint ownership in the final policy document as something built up collaboratively rather than seeing it as something imposed through ‘top-down’ project structures.

Finally, using an iterative discussion component also acts as an exploratory space where different options can be proposed, potential gaps in planning brought up, and compromises negotiated. This can be seen as a challenge for project management and organisation, but it also has a number of potential benefits that are worth considering beyond the StemBANCC case. Having a space for open discussion can bring to light issues and concerns of different partners that might otherwise not have found expression elsewhere and provides an opportunity for discussion and resolution of these issues prior to any disagreement that might arise at a more critical juncture in a project. Of course, such a potentially open-ended process requires careful time management strategies. Within StemBANCC an overall timeframe was set in advance by having an active BMDAC as a specified project deliverable with a fixed delivery date. Proportionality - ensuring the burden of a particular governance mechanism, in terms of time, effort and workload involved, does not outweigh its advantages and targeting - making sure the governance structure stays focused only on the issues it was designed to address and does not grow too broad and unwieldy are also important in keeping the process on track. Examples in creating the StemBANCC BMDA policy include implementing a 3 days turnaround time for the BMDAC to review requests for cell lines, balancing the desire to audit level 1 and 2 requests with the time costs and privacy concerns of consortium partners, and making sure that issues such as future costs were moved out of the materials and data access policy discussion and into other fora within the consortium. Ultimately, the arrangements reached in StemBANCC do not necessarily exemplify a universalisable best practice; but they do represent a model for making the best decisions for sharing materials and data within a particular SCR consortium at a given time, and based on the information available.
